# Canine chronic idiopathic rhinitis: management and outcome – a single‐centre retrospective observational study

**DOI:** 10.1111/jsap.70086

**Published:** 2026-01-16

**Authors:** P. M. N. Henry, R. Rizzo, Y. Tan, A. M. Boag, J. Del‐Pozo, G. A. Woods

**Affiliations:** ^1^ The University of Edinburgh Hospital for Small Animals Roslin UK; ^2^ Department of Infectious Diseases University of California, Davis, School of Veterinary Medicine Davis California USA

## Abstract

**Objectives:**

Canine chronic idiopathic rhinitis is a common cause of nasal disease in dogs but data reporting outcomes following treatment is lacking. The aim was to describe pre‐ and post‐referral management and outcomes of dogs diagnosed with canine chronic idiopathic rhinitis at a single referral centre.

**Materials and Methods:**

Retrospective review of medical records (signalment, clinical signs, treatments prior to referral, investigations, response to therapy and outcomes) of 75 client‐owned dogs diagnosed with canine chronic idiopathic rhinitis between December 2018 and 2023.

**Results:**

Forty‐one males and 34 females were included. Duration of clinical signs prior to referral was 104 days [3 to 1954] and only 11/75 dogs had not received antibiotics. Tomography revealed destructive rhinitis in 54/75 dogs. After referral, the most common first‐line treatment was non‐steroidal anti‐inflammatory drugs (46/75). Sixty‐seven cases were followed up for 237 days [14 to 1701]. Forty‐six, 25 and 13 dogs underwent a second, third and fourth treatment trial, respectively. Antibiotics were the most used second trial agent (25/46), and corticosteroids the most common third (16/25). Outcomes were available for 63 dogs. At final recorded contact, 14/63 cases were in remission, 38/63 were considered improved, 9/63 had static disease and 2/63 had worse disease. Three cases were euthanased due to canine chronic idiopathic rhinitis. Non‐steroidal anti‐inflammatory drugs were significantly associated with clinical improvement after initial treatment trial (OR: 3.0, 95% CI: 1.1 to 8.5; *P*=.04).

**Clinical Significance:**

Dogs diagnosed with canine chronic idiopathic rhinitis received variable therapies, including frequent antibiotics. Administration of non‐steroidal anti‐inflammatory drugs as first‐line treatment was associated with improved outcomes in this cohort of dogs.

## INTRODUCTION

Canine chronic idiopathic rhinitis (CCIR) is a common inflammatory condition which accounts for up to 66% of dogs presenting with chronic nasal signs (Pietra et al., 2010). CCIR can be a frustrating disease for both veterinarians and owners and directly impacts the quality of life of affected dogs (Greene et al., 2017). The clinical signs and imaging findings are nonspecific (Gianella et al., 2020; Lobetti, 2014; Plickert et al., 2014; Windsor & Johnson, 2006). Diagnosis requires extensive investigations and relies on documenting chronic inflammation on histopathology without an identifiable cause (Cohn, 2020). Inflammation is predominantly reported as lymphoplasmacytic (LP), but granulomatous, suppurative and eosinophilic inflammation occurs as well (Greene et al., 2017; Vangrinsven et al., 2023).

The pathogenesis of CCIR remains poorly understood (Wang et al., 2024). Previous publications have not found supporting evidence for several hypotheses (underlying *Bartonella*, *Alternaria*, *Cladosporium* or subclinical *Aspergillus* infection), and others are still being investigated (dental disease, aerodigestive disorders and host–microbiota interactions) (Gianella et al., 2020; Hawkins et al., 2008; Mercier et al., 2013; Peeters et al., 2007; Salas García et al., 2024; Stepaniuk & Gingerich, 2015; Wang et al., 2024). This uncertain pathogenesis results in dogs undergoing multiple treatment trials. A plethora of treatment options are described including antibiotics, corticosteroids, non‐steroidal anti‐inflammatory drugs (NSAIDs), desensitisation, diet trials and antihistamines (Cagnasso et al., 2021; Gianella et al., 2020; Hamsayamini et al., 2015; Lobetti, 2014; Windsor et al., 2004; Yamaya & Watari, 2015). Only five studies offer descriptions of the outcomes of dogs treated for CCIR (Burgener et al., 1987; Gianella et al., 2020; Kaczmar et al., 2018; Lobetti, 2014; Windsor et al., 2004) (Table S1). A single prospective pilot study compared the effectiveness of various treatment protocols and suggested that 3 weeks of non‐steroidal anti‐inflammatory drugs followed by 3 weeks of glucocorticoids was the most successful (Kaczmar et al., 2018). Thus, treatment trials likely reflect clinician’s preferences rather than evidence. With tightening antibiotic regulations and a desire to rationalise their use, there is a need for information regarding the effectiveness of current therapies, particularly the use of antimicrobials (Lappin et al., 2017).

The aim of this study was to describe the management and outcome of a population of dogs diagnosed with CCIR in a single referral centre. We hypothesised that dogs with CCIR would undergo multiple treatment trials, including with antibiotics not administered in line with recommendations inferred from the International Society for Companion Animal Infectious Diseases (ISCAID) guidelines on canine upper respiratory tract diseases, that clinical outcomes would be variable but reflect progressive disease, and that mortality would be low.

## MATERIALS AND METHODS

This study was a retrospective review of medical records of dogs diagnosed with CCIR at a Veterinary Teaching Hospital in the United Kingdom between December 2018 and December 2023. Records were retrospectively reviewed by the same author. The clinicopathology database was interrogated for: (1) histopathology reports including one or several specific key words (rhinitis, nasal or nostril), (2) combined bacterial and fungal culture reports and (3) final diagnosis (rhinitis, chronic rhinitis and lymphoplasmacytic rhinitis). CCIR was defined according to previous literature as the presence of chronic inflammatory changes on histopathology without an identifiable cause (Greene et al., 2017). Absence of underlying disease was based on the absence of mass lesion or fungal plaque on tomography and rhinoscopy, in addition to the absence of hyphae or findings suggestive of neoplasia on histopathology and, when available, on additional negative fungal culture. Dogs previously treated for aspergillosis, with evidence of acute (catarrhal) rhinitis on histology, with stenosis described on nasopharyngoscopy or diagnostic imaging, with oronasal fistulas, suspicion of foreign body or clinical signs compatible with gastro‐oesophageal reflux were excluded.

The dogs’ signalment (breed, body weight, body condition score, sex and neutering status), clinical signs and their duration, treatments prior to referral (number, nature and reported response), results from investigations (haematology, biochemistry, infectious disease screening where performed, tomography, rhinoscopy, histopathology and microbiology) and following treatments were extracted from the medical files.

Tomography images were acquired using a 64‐slice helical CT scanner (technical settings of 120 kV, adaptive mAs and slice thickness ranging from 0.6 to 1 mm for the head and 1.5 to 2 mm for the thorax) before and after administration of intravenous contrast (Omnipaque™ 350 mgI/mL, GE Healthcare AS, Oslo, Norway). Studies were analysed using an open‐source DICOM viewer (Horos™) and bone, soft tissue and pulmonary reconstruction algorithms with adjustments to window width and level as needed. Images were reviewed by a diagnostic imaging clinician working under the supervision of a board‐certified diagnostic imaging specialist for presence of dental disease (visible tartar build up, attrition, resorption, periodontitis, tooth root abscess or other significant findings), enlargement of locoregional lymph nodes (retropharyngeal or retromandibular larger than 5.5 mm in short axis), accumulation of fluid in the paranasal sinuses, destructive changes (graded as mild, moderate or marked), accumulation of fluid in the nose, presence of fluid within the airways (choanae, nasopharynx or trachea) and evidence of pneumonia when thoracic imaging was available (defined as lung lobe consolidation associated with air bronchograms or increased attenuation or soft tissue appearance of the lung parenchyma without loss of volume) (Belotta et al., 2022; Constantinescu et al., 2023).

Endoscopy was performed by internal medicine clinicians working under the supervision of board‐certified internal medicine specialists. Rhino‐pharyngoscopy was performed using flexible endoscopes (bronchoscope 5.3 mm × 70 cm reference AVB‐69HQ and gastroscope 6 mm × 105 cm reference AGVE‐69PQ, Huger Medical Instruments Co. Ltd, Shanghai, China; disposable bronchoscope 4.9 mm × 60 cm, Vathin Medical Instrument Co. Ltd., Xiangtan, China) and anterograde, as per clinician’s preference, using either the same flexible scope or a rigid one (multipurpose rigid telescopes 30° 2.7 mm × 18 cm and 5.5 mm × 23 cm, reference 64029BA and 63026U, Karl Storz, Tuttlingen, Germany). Biopsies were acquired at the attending clinician’s discretion, either blindly or endoscopically. Endoscopy reports were reviewed for the presence of mucus, hyperaemia, friable mucosa and prominent lymphoid follicles, but not for destructive changes.

Histopathology reports were classified as lymphoplasmacytic if displaying a typical mucosal or submucosal infiltrate with lymphocytes and plasma cells, or were reviewed by a board‐certified pathologist and classified as “other” type of inflammatory chronic rhinitis (OTICR) otherwise. Additional data that were recorded included the presence of other inflammatory infiltrates (active or neutrophilic, eosinophilic, granulomatous), whether sampling was unilateral or bilateral, whether diagnosis correlated between nostrils and the severity of inflammation (as stated on the report: mild, moderate or marked to severe). When severity was different between nostrils, the most severe was recorded for later analysis.

Infectious disease tests performed during investigations were reviewed, specifically bacterial and fungal culture of nasal biopsies and, when available, bronchoalveolar lavage cytology, bacterial culture and polymerase chain reaction (PCR) panel for lower airways infections. Bacterial cultures were performed on Columbia blood agar with 5% v/v defibrinated horse blood (E & O Laboratories, Bonnybridge, UK) at 37°C; one plate was incubated aerobically, and a second plate was incubated in an anaerobic jar using an AnaeroGen sachet (Oxoid, Basingstoke, UK). Bacterial identification and sensitivity testing were performed using Vitek2® following the manufacturer’s instructions (BioMérieux, Basingstoke, UK). Sensitivity testing was done using the Vitek2®AST‐GN97 and AST‐GP80 cards and applying appropriate Clinical and Laboratory Standards Institute‐based breakpoints. Fungal cultures were performed on Sabouraud Dextrose Agar (E&O, Bonnybridge, UK) at 37°C. Samples that were too small to immediately plate out were enriched in Nutrient broth (Oxoid, Basingstoke, UK) containing 5% horse serum (Sigma Aldrich, USA) overnight and plated out as described above.

When available, follow‐up assessments were based on clinical notes from following appointments, unprompted recorded communications from owners or from specific communications with the referring veterinarians (email). Outcomes were subjectively appraised by a single investigator and classified based on changes in frequency or intensity of clinical signs as worse (one or several clinical signs with increased intensity or frequency), static (overall no change reported or one sign better and one worse), improved (at least one clinical sign reported improved in intensity or frequency and no clinical sign worsening), or in remission if all medications had been discontinued and the clinical signs remained mild enough for the owners not to seek further treatment.

Data were organised in Microsoft Excel (Microsoft, USA) and statistical analyses performed in R (R version 4.4.1) using RStudio (version 2024.09.0+375). Normality was assessed by visual inspection of data and q‐q plots; the data were not normally distributed. Chi‐square test was used to compare independent categorical data, and the Mann–Whitney *U* test for continuous ones. Odds ratios are presented with their 95% confidence intervals (95% CIs). Data are presented as frequencies and percentages for qualitative variables and median [range] for quantitative variables. Statistical significance was defined as *P* < .05.

## RESULTS

### Case identification

Following removal of duplicates, 315 individual dog records were identified and screened for CCIR; of these, 75 remained after exclusion (Table 1). There were 34 females (six entire and 28 neutered) and 41 males (13 entire and 28 neutered). The median age was 8 years old [0.5 to 14] and the median body weight was 16.0 kg [2.0 to 72.7]. Assessment of breed distribution showed crossbreed dogs, springer spaniels, Border terrier, Chihuahua, husky, Lhasa apso, whippet, malamute and Dalmatian were over‐represented (Table 2).

**Table 1:** Summary of case recruitment outlining the exclusion criteria

	Histopathology *n* = 212	Microbiology *n* = 65	Diagnostic tag *n* = 38	Total *n* = 315
Included	75	0	0	75
Excluded				
No histopathology or inconclusive	1	2	38	41
Not nasal	16	59	0	75
Mass lesion on imaging	15	1	0	16
Neoplasia	54	0	0	54
Aspergillus	26	3	0	29
Other infectious causes (ehrlichiosis, M*ycoplasma* sp., *Bordetella bronchiseptica*)	4	0	0	4
Acute rhinitis, nasopharyngeal disease (stenosis, polyp), oronasal fistula and/or foreign body, no rhinoscopy	21	0	0	21

**Table 2:** Breed distribution of 75 dogs diagnosed with CCIR. Several breeds were significantly over‐represented

Breed	Frequency *n* (%)	Frequency *n* (%) attending the hospital over the study period	*P*‐value	Odds ratio	95% confidence interval
	*N* total = 75	*N* total = 24,003			
Cross breed*	12 (16)	1346 (5.6)	**1.1 × 10** ^**−4**^	3.2	1.7 to 5.9
Springer spaniel	6 (8)	701 (2.9)	.**01**	2.9	1.2 to 6.6
Border terrier	5 (7)	369 (1.5)	**3.5 × 10** ^**−4**^	4.6	1.8 to 11.4
Cocker	5 (7)	1606 (6.7)	.97		
Dachshund	4 (5)	509 (2.1)	.06		
Husky	4 (5)	186 (0.8)	**<10** ^**−5**^	7.2	2.6 to 20.0
Labrador	4 (5)	2759 (11.5)	.09		
Lhasa apso	4 (5)	302 (1.3)	.**002**	4.4	1.6 to 12.1
Whippet	4 (5)	219 (0.9)	**6.5 × 10** ^**−5**^	6.1	2.2 to 16.9
Malamute	3 (4)	44 (0.2)	**<10** ^**−5**^	23.9	7.3 to 79.1
Chihuahua	3 (4)	256 (1.1)	.**01**	3.9	1.2 to 12.5
West Highland white terrier	3 (4)	370 (1.5)	.09		
Border Collie	2 (3)	732 (3.0)	.83		
Dalmatian	2 (3)	98 (0.4)	.**002**	6.7	1.6 to 27.7
French bulldog	2 (3)	850 (3.5)	.67		
German shepherd dog	2 (3)	424 (1.8)	.57		
Other breeds	10**				

Significant *P*‐values (i.e. <. 05) are in bold.

* Including some intentional cross breed: four springer spaniel and cocker mix, two labrador and poodle mix and one cocker and poodle mix

** One of each: bichon, boxer, Cavalier King Charles, flat coat retriever, Jack Russell terrier, lurcher, miniature schnauzer, Newfoundland, saluki and Staffordshire bull terrier

### Clinical signs and physical examination

Clinical signs were mostly chronic, with a median duration prior to referral of 104 days [3 to 1954]. Sneezing (69/75, 92%) and nasal discharge (66/75, 88% of dogs, 46 bilateral and 20 unilateral) were the most frequent, but cough was also common (45/75, 60%). Discharge was purulent in most cases (45/66, 68%), occasionally mucoid or serous (14/66 and 6/66, respectively). Description of the discharge was not recorded in one case. Intermittent epistaxis was noted in 13 dogs (13/75) and three had nasal discolouration (3/75). The clinical signs and their frequency are summarised in Table 3. Dental disease (as stated on the medical report, often without specific description of changes) was reported in 28 dogs (28/75, 37%). Sixteen cases (16/75, 21%) were reported to have prominent or enlarged locoregional lymph nodes on physical examination, of which only six had concurrent dental disease.

**Table 3:** Clinical signs and their frequency in 75 dogs diagnosed with CCIR. Sneezing and nasal discharge were the most frequent

Sign	Frequency *n* = 75 (%)
Sneezing	69 (92)
Nasal discharge	66 (88)
Coughing	45 (60)
Reverse sneezing	38 (51)
Dental disease*	28 (37)
Upper respiratory tract noises	25 (33)
Gagging, retching	24 (32)
Epistaxis	13 (17)
Pawing at face	13 (17)
Halitosis	6 (8)

* Dental disease as reported on physical examination

### Histopathology findings

Forty‐five reports diagnosed lymphoplasmacytic rhinitis (LPR), and in 30, the final pathological diagnosis was chronic rhinitis. After review by a single investigator, 3/30 did not describe the typical mucosal or submucosal infiltrate with lymphocytes and plasma cells. These reports were further reviewed by a board‐certified pathologist (one showed a mixture of plasma cells and neutrophils, one plasma cells and eosinophils and one with submucosal oedema and infiltration with lymphocytes and neutrophils only) and were classified as OTICR.

Most cases had bilateral biopsies (62/75, 83%); the rest were unilateral. Of 62 bilateral samples, 54/62 (87%) returned identical diagnoses (albeit with different severity between nostrils in six cases). The eight remaining cases had different pathological findings between sides. All were diagnosed with chronic rhinitis in one nostril and in the other suppurative oedema (*n* = 3), normal tissue (*n* = 2), acute rhinitis (*n* = 1), submucosal oedema (*n* = 1) or LPR with eosinophilic infiltrate (*n* = 1).

Severity on histopathology was mild in 22/75, moderate in 40/75, marked to severe in 11/75 and not stated in two cases. An active or neutrophilic component was present in 56/75 (75%). Eosinophilic inflammation was present in seven cases (6 LPR and 1 OTICR), one of each: Border terrier, crossbreed, husky, Jack Russel terrier, Labrador retriever, malamute, Newfoundland. The severity of changes on histopathology was moderate (*n* = 4) or severe (*n* = 3). Cases with a neutrophilic or active component had significantly more severe changes on histopathology than those without (*P* = .001).

### Cranium tomography

Computed tomography (CT) of the head was performed in all dogs. Changes consistent with accumulation of fluid in the nasal cavities were present in 56/75 cases (75%). Destruction of turbinates was described in 54/75 cases (72%), unilateral in 20/54 cases (37%) and bilateral otherwise (34/54, 63%). Severity was mild in 38 cases (38/54, 70%), moderate in 13 (13/54, 24%) and marked in three cases (3/54, 6%). Duration of clinical signs was not significantly different between cases with moderate to marked turbinate destruction (*n* = 16, median 100 days [42 to 1714]) and less severe changes (*n* = 38, 87 days [3 to 1954], *P* = .65). Epistaxis was not significantly different between cases with less severe changes (9/38) and those with moderate to marked destructive rhinitis (2/16, *P* = .35). Fluid material was present in the paranasal sinuses in 34/75 cases (45%). Accumulation of fluid around the oropharynx or choanae (postnasal drip) was described in 27/75 cases (36%). Dental changes (other than oronasal fistula) were present on tomography in 69/75 (92%); specifically: visible tartar build up (45/69, 65%), periodontitis (54/69, 78%), attrition (23/69, 33%), tooth resorption (19/69, 28%), tooth root abscess without nasal communication (13/69, 19%) and tooth fracture (3/69, 4%). Enlargement of the locoregional lymph nodes was described in 43/75 dogs (57%), bilaterally in 34 cases and unilaterally in nine. Of the latter, lymphadenomegaly was contralateral to the main nasal lesion in four cases, on the same side in four more and unilateral right in the last case diagnosed with bilateral rhinitis.

### Thorax tomography and bronchoalveolar lavage (BAL)

A total of 66 dogs had tomography of the thorax. There was no evidence of pathology in half of the cases (33/66, 50%). The 33 remaining ones had fluid, mucous or frothy material in the trachea (*n* = 19), changes suggestive of pneumonia (*n* = 7) or both (*n* = 7). Cough was significantly more frequent in cases with thoracic changes on tomography than in cases without (27/33, 82% vs. 16/33, 48%; OR 4.8, 95% CI: 1.6 to 14.6, *P* = .004). However, CRP was not significantly higher (*P* = .08).

Bronchoalveolar lavage was performed in 20 cases, 15 diagnosed with thoracic changes on CT and five without CT changes but with a concurrent cough. Two were unsuccessful (non‐diagnostic cytology and no bacterial growth). Of the remaining 18, 16 documented neutrophilic or mixed inflammation, one eosinophilic inflammation and one mild hemosiderosis. Two samples revealed the presence of eosinophils; one showed a mild mixed neutrophilic and eosinophilic inflammation in a Newfoundland with eosinophilic rhinitis; the other showed a mixed cell to mildly eosinophilic infiltrate in a crossbreed dog without eosinophilic component on nasal histopathology. Bacterial culture was negative for 9/20 cases and returned mixed bacterial growth (*n* = 6), *Pasteurella canis* (*n* = 2) and one of each: *haemolytic E. coli*, *Sphingomonas paucimobilis* and *Clostridium histolyticum*. Further polymerase chain reaction (PCR) for specific airway pathogens (*Angiostrongylus vasorum*, *Crenosoma vulpis*, *Crenosoma parainfluenza*, *Crenosoma coronavirus*, canine adenovirus type 2, *Mycoplasma cynos* and *Bordetella bronchiseptica*) was conducted in 15 cases. All returned negative results. Ten cases with positive bacterial culture were treated with antibiotics (doxycycline *n* = 8 and marbofloxacin *n* = 2). The untreated dog had a light mixed growth of bacteria consistent with oral contamination.

### Rhinoscopy

Anterograde rhinoscopy and flexible retroflexed nasopharyngoscopy were performed in all cases. A complete endoscopy report was available for 53 cases, and a limited description of the nasal changes, but no description of nasopharyngoscopy, for 22. The most common changes during nasopharyngoscopy were mild to moderate hyperaemia (35/53, 66%) and accumulation of mucus in the choana (21/53, 40%). Prominent lymphoid follicles were less frequent (11/53, 21%). On anterograde rhinoscopy, bilateral changes were more frequent (69/75, 92%) than unilateral (6/75, 8%). Hyperaemia was the most common alteration of the nasal mucosa and was reported in 66/75 cases (88%). The presence of increased mucus was described in 48 cases (48/75, 64%) and friability in 30 cases (30/75, 40%).

### Bacterial and fungal culture of nasal biopsies

Bacterial culture of nasal tissue was positive in 32 cases, negative in 20 and not performed in 23. When positive, growth was light (*n* = 20), moderate (*n* = 8) or heavy (*n* = 4) and involved either a single organism (pure growth, *n* = 15) or multiple (mixed growth, *n* = 17). When identified (14/15), the pure growths were *Staphylococcus* sp. (*pseudintermedius*, *n* = 5 and *aureus*, *n* = 1), *Pasteurella canis* (*n* = 4), *Pseudomonas aeruginosa* (*n* = 2), *Streptococcus* sp. (*n* = 1) and *E. coli* (*n* = 1). Fungal culture was negative in 46 cases and not performed in 29.

### Treatment prior to diagnosis

**FIG 1:**
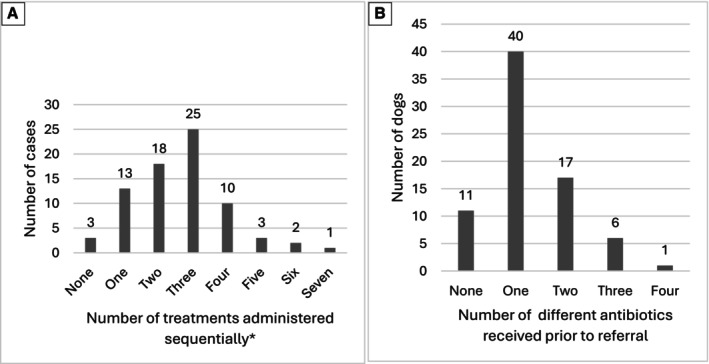
Histogram of the total number of different treatments received prior to referral (left) and number of different antibiotics (right) in a cohort of 75 dogs treated for CCIR. The use of multiple sequential treatments and multiple antibiotics was frequent. *Each antibiotic trial is considered as a separate treatment.

Treatments prior to referral were varied, numerous and often involving multiple concurrent agents (Table 4 and Fig 1). Antibiotic use was frequent (64/75 dogs, 85%), mainly in combination with other drugs but as sole therapy in 8/75 (11%). The most prescribed antibiotic was potentiated amoxicillin (52/75, 69%); doxycycline was used in 17 cases (17/75, 23%). Other types of antibiotics were also prescribed, including fluoroquinolone and third‐generation cephalosporins in 12 cases (12/75, 16%) (Table 4). Only 11 dogs did not receive antibiotics prior to referral and were either not treated (*n* = 1), received NSAIDs (*n* = 6) or corticosteroids (*n* = 2) or both sequentially (*n* = 1).

**Table 4:** Frequency of treatment administered prior to referral in 75 dogs diagnosed with CCIR. Most dogs received antibiotics prior to referral

Treatment	Frequency, *n* = 75
Antibiotics	
Amoxicillin and clavulanic acid	52*
Doxycycline	17*
Fluoroquinolones or third‐generation cephalosporins**	12*
Other types of antibiotics***	12*
Other treatments	
NSAIDs	36*
Corticosteroids	34*
Antihistamine	15*
Bromhexine	12*
Nasal flush	4*
Inhaled corticosteroids	2*

* The cases do not add up to 75 as many dogs received more than one antibiotic

** Specifically: marbofloxacin *n* = 6, enrofloxacin *n* = 4, pradofloxacin *n* = 1 and cefovecin *n* = 1

*** Specifically: clindamycin *n* = 6, oxytetracyclines *n* = 2, cefalexin *n* = 4, metronidazole *n* = 2 and tylosine *n* = 1

### Treatment after diagnosis

**FIG 2:**
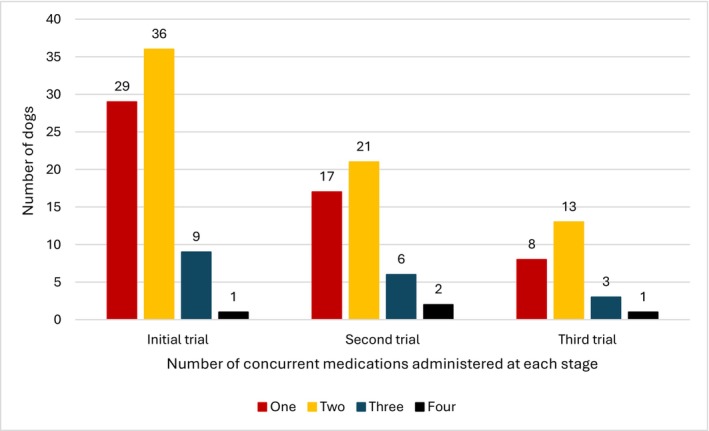
Histogram of the number of concurrent treatments administered after referral to 75 dogs diagnosed with CCIR at each sequential trial. Dogs received multiple sequential treatment trials, commonly involving one or two concurrent medications.

After referral, dogs were treated with between one and four concurrent medications (Fig 2). Initial treatment trials are summarised in Table 5 and detailed in Table S2. Median duration for the first treatment trial was 28 days [4 to 450]. NSAIDs were the most prescribed first‐line agent, administered to 46 dogs (meloxicam *n* = 43, firocoxib *n* = 2 and grapiprant *n* = 1; as sole agent *n* = 23 or combined with other drugs *n* = 23). Doxycycline was the second most common treatment, in 22/75 cases (29%), combined with another agent in all cases but one. Saline nasal flush at the time of endoscopy was reported in 34 cases (34/75, 45%). Four dogs had intra‐procedure instillation of clotrimazole while waiting for biopsy results and fungal culture (all negative for *Aspergillus* with no fungal plaque on rhinoscopy and confirmed as chronic rhinitis with no fungal hyphae on histopathology).

**Table 5:** Overall use of various medications as first‐line treatment of CCIR, in a cohort of 75 dogs, following referral. NSAIDs were the most used followed by doxycycline

Treatment	Frequency (*n* = 75)
Non‐steroidal anti‐inflammatory drug (NSAID)	46
Doxycycline	22
Corticosteroids per os	13
Inhaled corticosteroids	12
Hypoallergenic or hydrolysed diet	11
Other type of antibiotics	7*
Antihistamine	4
Miscellaneous	
Paracetamol	6
Fenbendazole	3
Gabapentin	3
Bromhexine	2
N‐acetyl cysteine	2
Nebulisation	1
Immunotherapy	1
Omeprazole + Metoclopramide	1

* Specifically: amoxicillin and clavulanic acid (*n* = 3), marbofloxacin (*n* = 2) and clindamycin (*n* = 2)

Sixty‐seven cases were followed up for 237 days [14 to 1701]. Forty‐six, 25 and 13 dogs underwent a second, third and fourth treatment trial, respectively (Table 6). The most common second treatment trial was a course of antibiotics (25/46 dogs, 54%), mainly using doxycycline (17/25, 68%). Of note, only two dogs underwent dental treatment, both as a second trial and alongside NSAID and antibiotics (one was later lost to follow‐up; the other required two further treatment trials). Third‐line treatments were dominated by corticosteroids (16/25, 64%, oral *n* = 12, inhaled *n* = 8, given in combination *n* = 4). Fourth trials were variable but included repeated antibiotic courses and inhaled corticosteroids. Other treatments not summarised in Table 6 and administered at various stages included immunosuppressive therapy with ciclosporin, allergy testing followed by desensitisation and the use of nasal drops (phenylpropanolamine, maropitant or eye drops containing sodium cromoglicate used off‐licence and repurposed as nasal drops).

**Table 6:** Summary of medications used as second, third and fourth treatment trials in a cohort of 75 dogs diagnosed with CCIR. Dogs received a variety of medication with no consensus

Treatment	Second trial *n* = 46	Third trial *n* = 25	Fourth trial *n* = 13
Antibiotics	25	8	7
Inhaled corticosteroids	16	8	6
Corticosteroids	15	12	4
NSAIDs	14	8	3
Hypoallergenic/hydrolysed diet	5	2	0
Antihistamine	3	4	0

### Outcomes

Through follow‐up, outcomes were available for 62 cases after initial treatment, 37 after the second trial, 19 after the third trial and eight after the fourth trial and are summarised in Table 7. Overall, at the time of last contact, outcomes were available for 63 dogs; 14 were in remission, the majority were improved (38/63, 60%), nine were reported static and two cases were worse. During the follow‐up period (237 days [14 to 1701]), 13 dogs died, of which three were euthanased for reasons directly relating to their nasal disease (two for unacceptable quality of life, with nasal disease reported as static and mildly improved at last contact, and one with unmanageable recurrent epistaxis reported as worse).

**Table 7:** Detailed response to treatment obtained after each treatment trial, at time of last contact and broken down per treatment after trial 1. Response to treatment was unpredictable, but most dogs eventually improved

	Worsened	Static	Improved	Remission (later relapse)	Total
Trial 1 (overall)	3	18	31	9 (1)	62
*Outcomes observed after Trial 1 – subclassified for the three main categories of treatment* *					
1. Antibiotics overall	1	10	10	3	24
Doxycycline	0	7	8	2	17
Others	1	3	2	1	7
2. NSAID	2	5	22	6 (1)	36
3. Corticosteroids overall**	2	8	7	2	19
Corticosteroids (oral)	1	7	3	1	12
Inhaled corticosteroids	2	2	4	1	9
Trial 2	7	3	20	4 (3)	37
Trial 3	0	6	12	0 (1)	19
Trial 4	0	0	7	1	8
*Overall – at last contact*	2	9	38	14	63

* The subclassified columns do not add up to the same number as the overall for trial 1 because many dogs received several concurrent treatments

** The subclassified columns do not add up to the same number, as some dogs had concurrent inhaled and per‐os corticosteroids

### Association between exposure to specific treatments and outcome

Administration of NSAIDs as initial treatment trial, compared to no administration of NSAIDs, was overall associated with outcome category (*P* = .02). Looking at sub‐groups (NSAID, antibiotics overall, doxycycline specifically and corticosteroids), NSAID at initial trial was positively associated with improved outcome (22/31 improved cases received NSAIDs but only 14/31 of the not improved ones, OR: 3.0, 95% CI: 1.0 to 8.5; *P* = .04) and negatively with static disease (only 5 of the 18 static cases had received NSAID, in contrast 31/44 of the non‐static cases had received NSAID, OR: 0.16, 95% CI: 0.0 to 0.5; *P* = .002). There was no significant association observed between other initial treatments assessed and outcomes, nor between these treatments given at later trials and outcomes.

### Association between positive bacterial culture and outcome

There was no significant association between outcome categories and the documentation of a positive or negative bacterial culture on nasal biopsies (Table 8, *P* = .81).

**Table 8:** Outcomes recorded in 75 dogs diagnosed with CCIR subclassified according to the presence/absence of bacteria on culture of nasal biopsies. Outcomes were not significantly different whether bacteria were present or absent

	Culture positive (*n* = 32)*	Culture negative (*n* = 20)*
Worse	1	1
Static	3	2
Improved	18	9
Remission	5	5

* The numbers of cases in each column do not add up to 32 and 20 because five and three cases with positive and negative bacterial culture, respectively, had no outcome recorded

### Association between destructive changes on tomography and outcome

There was no significant association between outcome categories and the severity of destructive changes on tomography (Table 9, *P* = .47).

**Table 9:** Outcomes recorded in 75 dogs diagnosed with CCIR subclassified according to the severity of tomographic changes. There was no correlation between severity of the changes and documented outcome

	Absent (*n* = 21*)	Mild (*n* = 38*)	Moderate to severe (*n* = 16*)
Worse	0	2	0
Static	1	7	1
Improved	12	17	9
Remission	5	6	3

* Cases do not add up to 21, 38 and 16, respectively, because outcomes were lacking for three cases with entire turbinates, six cases with mild and three cases with moderate to severe destruction

### Cases that entered remission

Breed distribution and selected specific findings from the cases that entered remission and the ones for which another outcome was recorded are available in Table S3.

### Treatment and outcomes of patients with eosinophilic rhinitis

Treatments and outcomes for the seven cases of eosinophilic rhinitis are detailed in Table S4. At the time of last contact, five dogs were alive (four improved and one static) and two had been euthanased for reasons relating to their disease (one malamute reported worse and one husky reported static). Response to corticosteroids (oral or inhaled) was not different whether eosinophilic infiltrate was present or not (2/5 eosinophilic rhinitis reported improved or in remission after initial treatment with corticosteroids vs. 7/14 dogs with non‐eosinophilic rhinitis, *P* = .7).

## DISCUSSION

This study provides a comprehensive review of the management of CCIR in a single referral centre in the United Kingdom. In keeping with our hypothesis, we discovered a frequent use of antibiotics, often not in line with recommendations that could be inferred from the ISCAID guidelines on canine infectious respiratory disease complex (Lappin et al., 2017). Antibiotics provide only minimal or temporary improvement in nasal signs by most likely reducing secondary bacterial colonisation rather than treating chronic rhinitis (Gianella et al., 2020; Kaczmar et al., 2018; Lobetti, 2014; Stepaniuk & Gingerich, 2015; Windsor & Johnson, 2006). Unfortunately, no specific guidelines exist for the treatment of nasal disease. However, the ISCAID guidelines discourage the use of antimicrobials for the treatment of upper respiratory tract disease, unless fever, lethargy and inappetence are present, together with mucopurulent discharge. If used, doxycycline is preferred as a first‐line treatment for its efficacy against *Mycoplasma cynos* (Lappin et al., 2017). In the present study, these specific clinical signs were not assessed but potentiated amoxicillin was the most prescribed antibiotic prior to referral. The inappropriate use of antimicrobials is associated with increased risk of antibiotic‐related side effects and selection of resistance with little to no benefit to the patient (Shea et al., 2011).

Doxycycline was the most prescribed antibiotic following referral. This likely reflects adherence to the ISCAID guidelines. An alternative explanation for the use of doxycycline is its immunomodulatory properties which have been demonstrated in human medicine, in multiple animal models, and at sub‐antimicrobial doses in dogs (Kim et al., 2016; Park et al., 2020; Szatmári & van Geijlswijk, 2022). The mechanism of action is incompletely understood but likely includes inhibition of T cell proliferation and production of proinflammatory cytokines and enzymes as well as interactions with polynuclear neutrophils (Park et al., 2020; Rieder et al., 2024). Regardless, treatment with doxycycline was not associated with outcome categories in the present cohort of dogs. Seven dogs received other antibiotics as initial treatment trial following referral, of which only three were justified by evidence of aspiration pneumonia. Unfortunately, the retrospective nature of the study precludes assessing the clinical reasoning behind these choices, but it highlights the need for critical assessment of the pertinence of antibiotic treatment for both primary care and referral clinicians.

The limited evidence for antibiotic treatment in the management of CCIR questions the need for bacterial culture of nasal biopsies. Positive bacterial cultures are common in cases of CCIR, but like in the present work, the bacteria isolated are thought to mainly represent normal nasal flora (Lobetti, 2014; Tress et al., 2017; Windsor & Johnson, 2006). In this cohort of dogs, last recorded outcomes were not significantly different between cases with positive and negative bacterial culture (Table 8). Additionally, results of bacterial culture are site specific and the cultured colonies may not represent the pathogenic agent based on discrepancies between next generation sequencing and standard culture (Abramson et al., 1976; Niedenführ et al., 2024; Vangrinsven et al., 2023). Thus, bacterial culture of nasal biopsies appears of minimal diagnostic value, except in systematically unwell cases where significant secondary bacterial overgrowth is suspected (Lappin et al., 2017; Tasker et al., 1999). Similarly, bacterial cultures risk identifying commensal multi‐drug‐resistant bacteria which could precipitate the unnecessary use of second‐ or third‐line antibiotics. This highlights that pre‐analytical consideration (diagnostic stewardship) is as critical as antibiotic stewardship (National Academies of Sciences, Engineering, and Medicine et al., 2023).

In this cohort of dogs, Border terrier, Chihuahua, cross‐breed, Dalmatian, husky, Lhasa apso, malamute, springer spaniels and whippet were over‐represented. This contrasts with previous literature that only reported German shepherd dogs, Yorkshire terrier or more generally dolichocephalic dogs to be predisposed to CCIR (Lobetti, 2014; Meler et al., 2008; Windsor et al., 2004). Interestingly, two dogs with eosinophilic rhinitis belonged to breeds predisposed to eosinophilic bronchopneumopathy (Alaskan malamute and Siberian husky), but only one had a BAL which did not document eosinophilic inflammation (Clercx et al., 2000).

Most dogs had a neutrophilic infiltration on histopathology, and the presence of neutrophils was associated with increased severity of reported histopathological changes. This is expected since histopathology scoring of the inflammation relies on quantification of inflammatory cells, including neutrophils and eosinophils (Furtado & Constantino‐Casas, 2013). Neutrophils are commonly involved with acute inflammation but also contribute to chronic inflammation with debated beneficial and damaging effects (Herrero‐Cervera et al., 2022). The latter has been documented in equine upper airway disease, where the severity of clinical signs correlates with the severity of airway neutrophilia (Bullone et al., 2018). The association of an eosinophilic infiltrate with the severity of the rhinitis changes on histopathology was not assessed statistically due to the size of the sample (*n* = 7). Not all cases underwent bilateral nasal sampling, but in eight, the histopathological findings were different between nostrils. This has previously been reported and may prompt clinicians to pursue bilateral nasal biopsy sampling (Windsor et al., 2004).

Tomography revealed destructive changes in 54/75 (72%) of cases. The frequency of destructive changes is highly variable in previous literature (0% to 70%) and likely accounts for the methods of screening (radiographs vs. tomography) (Kaczmar et al., 2018; Windsor & Johnson, 2006). Marked and severe turbinate destruction are commonly described in fungal rhinitis, whereas milder changes are expected with CCIR (Lefebvre et al., 2005). In the present study, 16/54 (30%) cases had moderate (*n* = 13) or marked (*n* = 3) destruction on CT, of which none had evidence of plaque on rhinoscopy, and 12 had negative aspergillus cultures (not performed in four cases with moderate destruction). Four of these dogs (three with moderate destruction, one with marked) underwent instillation of clotrimazole at the time of rhinoscopy and were discharged with additional treatments (NSAID alone; NSAID and doxycycline; NSAID, inhaled corticosteroids and hypoallergenic food, or oral corticosteroids and hypoallergenic food). Two had depigmentation of the nose on physical examination, but none had a history of epistaxis; all had negative fungal culture, and one had further evidence of negative aspergillosis (negative Grocott–Gomori’s methenamine silver stain). Two were lost to follow‐up; the other two were reported to be improved but required a second treatment trial with corticosteroids. The diagnosis of sino‐nasal aspergillosis can be challenging and dogs are typically diagnosed when they display either compatible clinical signs, turbinate destruction and fungal plaques or, if fungal plaques are not visible, have at least one positive result from ancillary diagnostic tests (fungal culture, serology, or PCR) (Prior et al., 2024). None of these four dogs met the criteria for the diagnosis of aspergillosis. While acknowledging the limitations of the investigations undertaken and the lack of follow‐up for two cases, this suggests that inflammatory rhinitis remains a plausible differential diagnosis, even with extensive turbinate lysis.

Tomography of the thorax revealed changes suggestive of pneumonia in 14/66 (21%) of dogs, most of which had a cough (11/14). Nine underwent BAL, of which six returned a septic neutrophilic cytology and positive bacterial culture. Although we cannot be certain that the nasal discharge caused pneumonia, most of these dogs were referred for investigation of chronic nasal signs and only one primarily for coughing. The present study is, to the authors’ knowledge, the first to describe a rhinitis‐bronchopneumonia syndrome presumed secondary to postnasal drip with aspiration in dogs other than Irish Wolfhounds (Clercx et al., 2003). In human medicine, people diagnosed with chronic rhinosinusitis are at increased risk of developing pneumonia (McQuitty et al., 2019; Özbay & Arslan, 2002; Wee et al., 2022). In this cohort of dogs, the frequency of fluid in the paranasal sinuses was not significantly different between dogs with and without pneumonia (8/14 vs. 23/52, *P* = .39). Our findings suggest that imaging of the thorax may be of value in dogs with chronic nasal disease, particularly if coughing is also reported.

Tomography revealed dental disease in a majority of dogs (68/75, 92%), of which less than half had been noted on physical examination. Odontogenic infection is common in cases of CCIR and should be carefully evaluated as appropriate dental treatment may resolve the histological changes in some cases (Stepaniuk & Gingerich, 2015). In the present study, two dogs underwent dental treatment as part of the management of their chronic rhinitis and were reported improved. This highlights the importance of a multidisciplinary approach to CCIR, with dental charting, probing and ideally specific imaging (dental radiographs). However, further studies are needed to ascertain the extent of improvement that can be expected from dental treatment alone.

The literature describing the treatments and outcomes of dogs with CCIR is limited to five studies, totalling 65 dogs (Table S1). Although immunosuppressive doses of corticosteroids were initially reported as effective, the most robust evidence comes from the study of Kaczmar et al., suggesting that sequential treatment with NSAIDs followed by corticosteroids was the most successful (*n* = 10 dogs, based on improvement in clinical signs, endoscopy and histopathology signs) (Kaczmar et al., 2018). Later studies failed to unveil significant associations between treatment strategies and outcomes (Gianella et al., 2020). The present study echoes these findings, describing multiple treatment trials with unpredictable responses and suggesting, as per previous literature, that administration of NSAIDs as first‐line treatment could be beneficial (Kaczmar et al., 2018). Although only 14 dogs entered remission, many cases improved with treatment.

An association between rhinitis and gastro‐oesophageal reflux disorders (GERDs) has been described in human medicine but remains controversial. While allergic rhinitis appears to predispose to GERD (Feng et al., 2021), a growing body of evidence suggests that GERD has a role in the development of chronic rhinosinusitis (Finocchio et al., 2021). Aerodigestive disorders are increasingly reported in veterinary medicine and are particularly common in brachycephalic breeds (Petchell et al., 2022). However, the co‐existence of CCIR with gastrointestinal disorders has only recently been described and its relationship remains unclear (Gianella et al., 2020). In the study by Gianella et al., dogs diagnosed with CCIR were investigated for concurrent gastrointestinal clinical signs (13/25) and endoscopic changes (22/25). Interestingly, 7/22 dogs were treated strictly for their gastrointestinal signs with either proton pump inhibitors and/or H2‐antagonists (*n* = 4) or hydrolysed diet (*n* = 3) and experienced a remission of their rhinitis (*n* = 2) or a marked improvement (*n* = 2). Although these findings were seemingly supportive of an aerodigestive aetiology for these dogs’ CCIR, the study did not demonstrate a definitive causative association between these two common inflammatory disorders. Another study reports an additional case of rhinitis and gastrointestinal disease (Salas García et al., 2024). However, this case had a relapse of rhinitis despite a persistent improvement in gastrointestinal signs, making aerodigestive disorders an unlikely aetiology (Salas García et al., 2024). In the present study, a single dog was suspected to have silent regurgitation resulting in chronic rhinitis. The chronic rhinitis persisted despite treatment with omeprazole and metoclopramide but improved after administration of doxycycline, making an aerodigestive aetiology unlikely. Regardless, these studies suggest a potential relationship between CCIR and chronic gastrointestinal disorders that warrants further research.

The present study has multiple limitations mainly pertaining to its retrospective nature including a limited number of cases, non‐standardised investigations or treatments and potential incomplete or inaccurate records. Cases had concurrent diseases that could have influenced the treatment of their rhinitis. Furthermore, infectious disease testing was not systematic due to the low prevalence of pathogens associated with nasal inflammation (leishmaniosis, ehrlichiosis, cryptococcosis) in the United Kingdom (Barbry et al., 2019; O’Neill et al., 2024; Silvestrini et al., 2023). For consistency, the outcomes were subjectively appraised by a single investigator, but assessment was based on recorded communications describing the owners’ perspective. This introduces a bias, as over time, owners accept the chronicity of the disease and signs that were initially considered as severe may become more tolerable. Although still owner‐based, the use of the SNIFLD questionnaire to grade the severity of nasal disease could provide a more accurate/consistent assessment of the dogs’ response to treatment (Greene et al., 2017).

In conclusion, dogs diagnosed with CCIR received variable therapies with unpredictable responses. The use of antibiotics is frequent and seemingly justified in only a small proportion of dogs diagnosed with CCIR and concurrent pneumonia. A consensus on the treatment of CCIR is lacking, but administration of NSAIDs as first‐line treatment was associated with improved outcomes in this cohort of dogs.

## Author contributions

**P. M. N. Henry:** conceptualization, data curation, formal analysis, investigation, methodology, project administration, visualization, writing original draft preparation. **R. Rizzo:** data curation, investigation, writing review and editing. **Y. Tan:** investigation, supervision, validation, writing review and editing. **A. M. Boag:** formal analysis, supervision, validation, writing review and editing. **J. Del‐Pozo:** investigation, supervision, validation, writing review and editing. **G. A. Woods:** conceptualization, supervision, validation, writing review and editing.

## Conflict of interest

No conflict of interest to report.

## Ethical approval

This study was approved by the Veterinary Ethical Review Committee of the Royal (Dick) School of Veterinary Studies (The University of Edinburgh Easter Bush Veterinary Centre Roslin, Midlothian EH25 9RG) under VERC reference 176.22.

## Supporting information


**Table S1.** Summary of previous publications describing the outcomes of dogs treated for CCIR.

**Table S2.** Detailed breakdown of treatments implemented following referral in 75 dogs diagnosed with CCIR.

**Table S3.** Selected findings in a cohort of 75 dogs diagnosed with CCIR subclassified according to whether remission was achieved or not.

**Table S4.** Detailed treatments and outcomes for seven cases diagnosed with eosinophilic rhinitis.

## Data Availability

The data that support the findings of this study are available on request from the corresponding author. The data are not publicly available due to privacy or ethical restrictions.
