# Supplementary material for: Canine chronic idiopathic rhinitis: management and outcome – a single‐centre retrospective observational study
Source: J Small Anim Pract. 2026 Jan 16;67(5):460–70. doi: 10.1111/jsap.70086 (PMC13136053; doi:10.1111/jsap.70086)
Supplement: Supplementary file 1 — Table S1. Summary of previous publications describing the outcomes of dogs treated for CCIR. [file JSAP-67-460-s001.docx]

Supplementary Table 1: Summary of previous publications describing the outcomes of dogs treated for CCIR.

| **Author** | **Type of study** | **Number of cases for which outcome was described**  **n = 65** | **Treatment** | **Outcome** |
| --- | --- | --- | --- | --- |
| **Gianella et al. 2020** | Prospective longitudinal | 22 / 25 | Group 1: Corticosteroids + antibiotics +/- mucolytic (n = 5)  Group 2: PPI +/- H2-antagonists +/- hydrolyzed diet (n = 7)  Group 3: Corticosteroids + antibiotics + H2-antagonists + hydrolyzed diet (n = 10) | Remission 2/5, improved 2/5, relapse 1/5  Remission 4/7, improved 3/7  Remission 2/10, improved 2/10, persistent 4/10, relapsed 1/10, worse 1/10 |
| **Kaczmar et al. 2018** | Prospective, randomized, control | 20 / 20 | Group 1: Tapering dose of per-os corticosteroids 6 weeks (n = 5)  Group 2: Meloxicam 3 weeks then corticosteroids 3 weeks (n = 5)  Group 3: Meloxicam 6 weeks (n = 5)  Group 4: Placebo 6 weeks then most effective treatment (n = 5) | All improved  Group 2 had the best outcome** |
| **Lobetti et al. 2014** | Retrospective case series | 14 / 33 | Allergy testing and desensitisation therapy (n=3)  Corticosteroids per-os (1 mg/kg SID) 7–10 days, then tapered and stopped +/- concurrent ciclosporin (5 mg/kg SID) (n= 11) | Remission  Improved |
| **Windsor et al. 2004** | Retrospective case series | 5 / 37 | Amoxicillin and clavulanic acid + corticosteroids per-os (n = 3)  Anti-histamine (n = 1)  Anti-histamine + inhaled corticosteroids (n = 1) | Improved*  Improved  Improved |
| **Burgener et al. 1987** | Retrospective case series | 4 / 5 | Corticosteroid per-os (2 mg/kg SID) | Improved |

* one of these dogs sneezed out a grass seed 2 months later and entered remission.

** based on improvement in clinical signs, endoscopy and histopathology signs

PPI: proton pomp inhibitor, SID: once daily
