# Supplementary material for: Canine chronic idiopathic rhinitis: management and outcome – a single‐centre retrospective observational study
Source: J Small Anim Pract. 2026 Jan 16;67(5):460–70. doi: 10.1111/jsap.70086 (PMC13136053; doi:10.1111/jsap.70086)
Supplement: Supplementary file 2 — Table S2. Detailed breakdown of treatments implemented following referral in 75 dogs diagnosed with CCIR. [file JSAP-67-460-s003.docx]

Supplementary Table 2: Detailed breakdown of treatments implemented following referral in 75 dogs diagnosed with CCIR.

| **Treatment** | **Frequency,**  **n = 75** | **Median duration in days (n)** | **Min – max** |
| --- | --- | --- | --- |
| ***Single agent:*** | ***29*** |  |  |
| - Non-steroidal anti-inflammatory | 23 | 28 (17) | 4 – 128 |
| - Corticosteroids per-os | 3 | 14 (3) | 14 – 28 |
| - Doxycycline | 1 | 120 (1) | x |
| - Other type of antibiotics | 1 | 42 (1) | x |
| - Inhaled corticosteroids | 0 |  |  |
| - Miscellaneous | 1^a^ | 28 (1) | x |
| - Hypoallergenic or hydrolysed diet | 0 |  |  |
| - Anti-histamine | 0 |  |  |
| ***Two agents:*** | ***36*** |  |  |
| - NSAID + Doxycycline | 7 | 28 (6) | 21 – 42 |
| - NSAID + Diet | 4 | 21 (4) | 14 – 28 |
| - NSAID + Inhaled cortico. | 3 | 71 (2) | 21 – 120 |
| - NSAID + Other type of antibiotics | 2 | 21 (2) | 7 – 35 |
| - NSAID + Anti-histamine | 2 | 21 (1) | x |
| - Doxycycline + Misc. | 5^c^ | 28 (5) | 21 – 42 |
| - Doxycycline + Cortico. PO | 2 | 9 (2) | 7 – 10 |
| - Doxycycline + Inhaled cortico. | 1 | 14 (1) | x |
| - Doxycycline + Anti-histamine | 1 | N/A | x |
| - Doxycycline + Diet | 1 | 28 (1) | x |
| - Other type of antibiotics + Misc. | 2^d^ | 39 (2) | 21 – 56 |
| - Other type of antibiotics + Cortico. PO | 1 | 14 (1) | x |
| - Cortico PO + Misc. | 2^e^ | 14 (2) | 7 - 21 |
| - Cortico PO + inhaled cortico | 1 | 21 (2) | x |
| - Cortico PO + Diet | 1 | 14 (1) | x |
| - Inhaled cortico + Misc | 1^f^ | 60 (1) | x |
| ***Three agents:*** | ***9*** |  |  |
| - NSAID + Doxycycline + Misc. | 1^h^ | 21 (1) | x |
| - NSAID + Doxycycline + Inhaled cortico. | 1 | x | x |
| - NSAID + Diet + Inhaled cortico. | 1 | 14 (1) | x |
| - NSAID + Diet + Misc. | 1^i^ | 70 (1) | x |
| - NSAID + Anti-histamine + Inhaled cortico. | 1 | 210 (1) | x |
| - Doxycycline + Cortico. PO + Misc. | 1^j^ | x | x |
| - Doxycycline + Inhaled cortico. + Misc. | 1^k^ | x | x |
| - Other type of antibiotics + Diet + Inhaled cortico. | 1 | 14 (1) | x |
| - Cortico PO + Diet + Misc. | 1^l^ | 14 (1) | x |
| ***Four agents:*** | ***1*** |  |  |
| - Diet + Cortico. PO + Inhaled cortico. + Misc. | 1^k^ | 14 (1) | x |

^a^ This dog underwent copious nasal flush and was discharged with gabapentin.

^c^ Additional medications included paracetamol (n = 3), bromhexine (n = 1) and fenbendazole (n = 1).

^d^ One received additional saline nebulisation and the other received paracetamol.

^e^ One received gabapentin and paracetamol, the other received omeprazole and metoclopramide.

^f^ This dog received additional paracetamol.

^h^ This dog underwent nasal flush and received additional N-acetyl cysteine.

^i^ This dog also received bromhexine and paracetamol.

^j^ This dog underwent nasal flush and received additional N-acetyl cysteine.

^k^ This dog was also treated with fenbendazole.

^l^ This dog underwent immunotherapy.

N/A not available, NSAID: non-steroidal anti-inflammatory, Cortico: corticosteroids, PO: per-os, Misc: Miscellaneous.
