# Supplementary material for: Canine chronic idiopathic rhinitis: management and outcome – a single‐centre retrospective observational study
Source: J Small Anim Pract. 2026 Jan 16;67(5):460–70. doi: 10.1111/jsap.70086 (PMC13136053; doi:10.1111/jsap.70086)
Supplement: Supplementary file 3 — Table S3. Selected findings in a cohort of 75 dogs diagnosed with CCIR subclassified according to whether remission was achieved or not. [file JSAP-67-460-s002.docx]

Supplementary Table 3: Selected findings in a cohort of 75 dogs diagnosed with CCIR subclassified according to whether remission was achieved or not.

| Findings | Remission (n = 14) | Not in remission (n = 49) |
| --- | --- | --- |
| **Breed** |  |  |
| - *German shepherd dog* | 2 | 0 |
| - *Labrador retriever* | 2 | 2 |
| - *Boxer* | 1 | 0 |
| - *Chihuahua* | 1 | 2 |
| - *Cross breed* | 1 | 10 |
| - *Dalmatian* | 1 | 0 |
| - *Flat coat retriever* | 1 | 0 |
| - *French bulldog* | 1 | 0 |
| - *Husky* | 1 | 2 |
| - *Miniature schnauzer* | 1 | 0 |
| - *Saluki* | 1 | 0 |
| - *Springer spaniel* | 1 | 5 |
| - *Other breeds* | 0 | 27 |
| **Duration of clinical signs (days)** | 120 [3 – 584] | 89 [5 – 1954] |
| **Treatments before diagnosis** | 2 [1 – 4] | 3 [0 – 7] |
| **Severity of histopathology changes** |  |  |
| - *Mild* | 4 | 15* |
| - *Moderate* | 7 | 29 |
| - *Severe* | 3 | 4 |
| **Destructive changes on CT** | 10/14 | 36/49 |
| - *Mild* | 6 | 26 |
| - *Moderate* | 1 | 10 |
| - *Marked* | 2 | 0 |
| **Other findings on CT:**   - *Fluid in the nasopharynx* - *Fluid in the trachea* - *Lung changes suggestive of pneumonia* | 1/13  1/13  2/13 | 22/49  21/44**  10/44** |
| **C Reactive Protein** | 4/14 | 17/49 |
| (mg/L) | 5.7 [2.5 – 15] | 2.5 [2.5 – 34] |

* The numbers do not add-up to 49 because severity of the changes on histopathology was not described in one case.

** Five cases did not undergo thoracic imaging.
