# Supplementary material for: Canine chronic idiopathic rhinitis: management and outcome – a single‐centre retrospective observational study
Source: J Small Anim Pract. 2026 Jan 16;67(5):460–70. doi: 10.1111/jsap.70086 (PMC13136053; doi:10.1111/jsap.70086)
Supplement: Supplementary file 4 — Table S4. Detailed treatments and outcomes for seven cases diagnosed with eosinophilic rhinitis. [file JSAP-67-460-s004.docx]

Supplementary Table 4: Detailed treatments and outcome for seven cases diagnosed with eosinophilic rhinitis.

|  | Trial 1 | Outcome 1 | Trial 2 | Outcome 2 | Trial 3 | Outcome 3 | Trial 4 | Outcome 4 | Last contact |
| --- | --- | --- | --- | --- | --- | --- | --- | --- | --- |
| Case 1 | Doxycycline  NSAIDs | Static | Antibiotics  NSAIDs | Worse | - | - | - | - | Euthanasia  worse |
| Case 2 | Cortico (PO)*  Gabapentin | Static | - | - | - | - | - | - | Euthanasia  static |
| Case 3 | Cortico (PO) | Static | Doxycycline  Cortico (PO) | N/A | Cortico (PO)  Ciclosporin | N/A | - | - | Static |
| Case 4 | Inhaled cortico | Improved | Inhaled cortico  Doxycycline | Worse | Inhaled cortico  Cortico (PO) | Improved | Desensitization | Improved | Improved |
| Case 5 | Doxycycline  Inhaled cortico | Static | Antibiotics  NSAIDs | Worse | Cortico (PO)  Hydrolysed diet | Improved | Inhaled cortico. | Improved | Improved |
| Case 6 | Clotrimazole  Cortico (PO)  Hydrolysed diet | Improved | Cortico (PO)  Inhaled cortico | N/A | - | - | - | - | Improved |
| Case 7 | NSAIDs | Improved | Antibiotics  NSAIDs  Dental treatment | Improved | Cortico (PO)  Inhaled cortico | Improved | Desensitization | N/A | Improved |

Abbreviations: cortico stands for corticosteroids, PO for per-os and N/A for not available.
